# Pulse-Wave-Pattern Classification with a Convolutional Neural Network

**DOI:** 10.1038/s41598-019-51334-2

**Published:** 2019-10-17

**Authors:** Gaoyang Li, Kazuhiro Watanabe, Hitomi Anzai, Xiaorui Song, Aike Qiao, Makoto Ohta

**Affiliations:** 1 Institute of Fluid Science, Tohoku University, 2-1-1, Katahira, Aoba-ku, Sendai, Miyagi 980-8577 Japan; 2 Graduate School of Biomedical Engineering, Tohoku University, 6-6 Aramaki-aza-aoba, Aoba-ku, Sendai, Miyagi 980-8579 Japan; 3 Department of Radiology, Taishan Medical University, No.619 Greatwall Road, Daiyue District, Taian, Shandong 271000 China; 4 College of Life Science and Bioengineering, Beijing University of Technology, No.100, Pingleyuan, Chaoyang District Beijing, 100022 China; 5 ELyTMaX UMI 3757, CNRS–Université de Lyon–Tohoku University, Sendai, Japan

**Keywords:** Biomedical engineering, Computational science

## Abstract

Owing to the diversity of pulse-wave morphology, pulse-based diagnosis is difficult, especially pulse-wave-pattern classification (PWPC). A powerful method for PWPC is a convolutional neural network (CNN). It outperforms conventional methods in pattern classification due to extracting informative abstraction and features. For previous PWPC criteria, the relationship between pulse and disease types is not clear. In order to improve the clinical practicability, there is a need for a CNN model to find the one-to-one correspondence between pulse pattern and disease categories. In this study, five cardiovascular diseases (CVD) and complications were extracted from medical records as classification criteria to build pulse data set 1. Four physiological parameters closely related to the selected diseases were also extracted as classification criteria to build data set 2. An optimized CNN model with stronger feature extraction capability for pulse signals was proposed, which achieved PWPC with 95% accuracy in data set 1 and 89% accuracy in data set 2. It demonstrated that pulse waves are the result of multiple physiological parameters. There are limitations when using a single physiological parameter to characterise the overall pulse pattern. The proposed CNN model can achieve high accuracy of PWPC while using CVD and complication categories as classification criteria.

## Introduction

**Figure 1:**
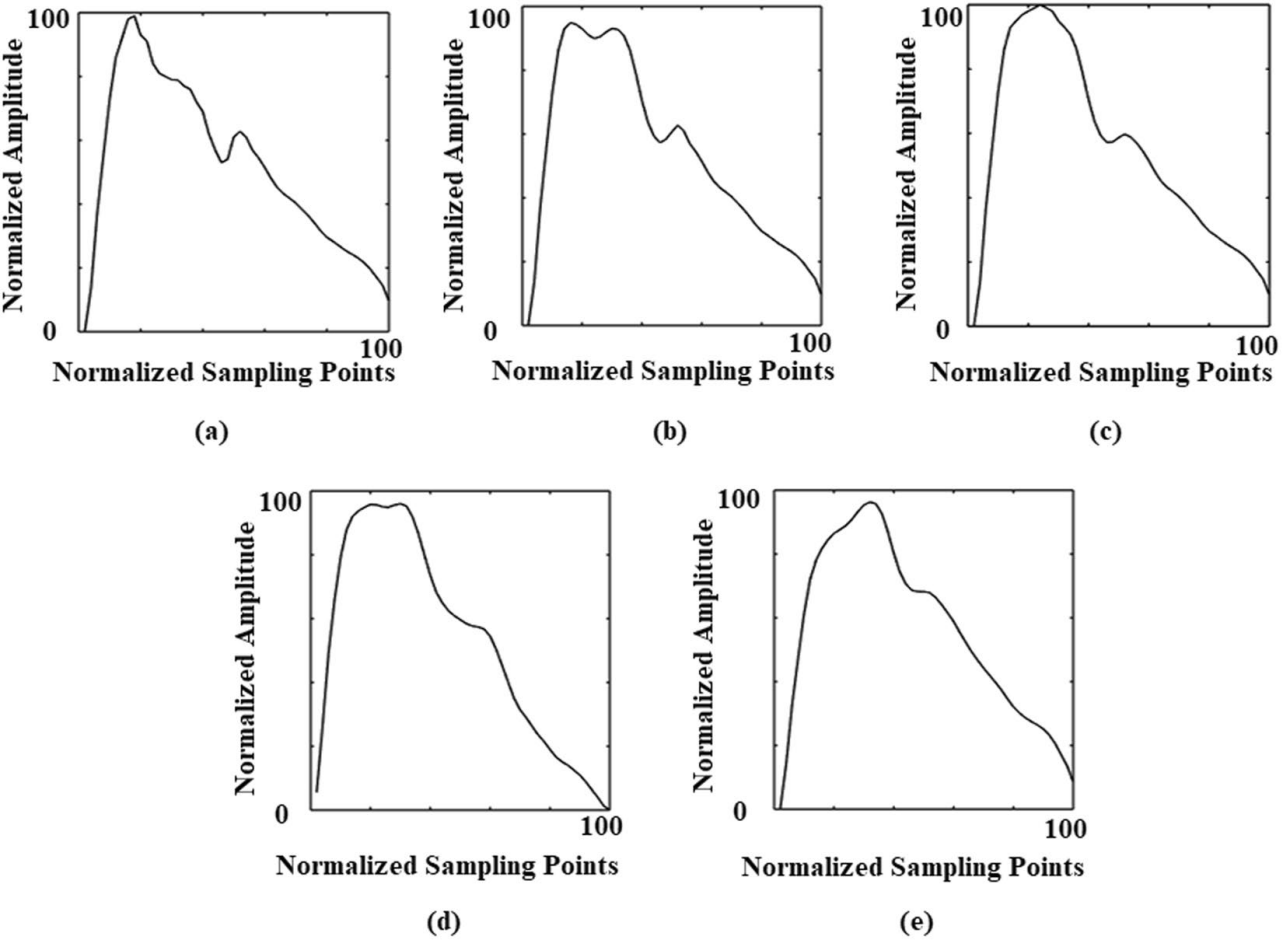
According to previous studies’ classification criteria, we show five pulse waves that exhibit a taut pulse pattern, which involves a pulse with a high second peak (local time shifting), as follows: **(a**) typical taut pulse, (**b**) taut pulse with high tidal wave and (**c**) taut pulse with tidal wave merged with percussion wave^9^. With the help of medical doctors, (**d**) and (**e**) were extracted from our database. Although (**a–e**) all feature a taut pulse pattern, there are still differences in some local waveform characteristics. In addition, the subject of (**d**) suffered from hyperlipidaemia, while the subject of (**e**) suffered from atherosclerosis. This shows that, under the previous classification criteria, a single pulse pattern might correspond to many disease categories.

Pulse waves contain a large quantity of pathological and physiological information^1,2^. Pulse-wave characteristics are closely related to diseases (hypertension, type 2 diabetes, atherosclerosis, etc.), especially cardiovascular diseases (CVD) and physiological parameters [pulse-wave velocity, cardio-ankle vascular index (CAVI), blood pressure, etc.]^3,4^. Therefore, pulse analysis is extensive used in cardiovascular function assessment and non-invasive early diagnosis of cardiovascular disease and related complications^5^. TCPD (Traditional Chinese Pulse Diagnosis) refers to the diagnosis of diseases via traditional Chinese medical practices by feeling the change in pulse at the patient’s wrist, which is highly dependent on the doctor’s skill and experience^6^. Computer-aided analysis has made some achievements in pulse diagnosis, especially in pulse-wave-pattern classification (PWPC). For example, Wang *et al*. divided 407 sets of pulse data into five pulse patterns by using a Bayesian network based on six pulse parameters: depth, width, length, frequency, rhythm and strength (84% successful classification rate)^7^. Moreover, Xu *et al*. divided 320 sets of pulse data into 16 pulse patterns by using a fuzzy neural network based on differences in pulse shapes, widths, positions and some specific local parameters (90% successful classification rate)^8^. However, the diverse morphology of pulse waves remains a difficulty for PWPC, which may lead to problems such as waveform local time shifting, as shown in Fig. 1^9^. In addition, the classification criteria of these studies are based on TCPD theory, which means that a pulse pattern may correspond to a variety of disease categories, as shown in Fig. 1^10^. It also leads to a decrease in the clinical practicality of pulse-based diagnosis. Thus, in this study, we selected new classification criteria—that is, the CVD and complication categories and the clinical physiological parameters—with the aim of developing a practical PWPC method with a high classification rate.

With the research and development of deep learning, various of neural network structures have been designed for signal processing. Recurrent neural network (RNN)^11^, based on its internal memory, is used to process arbitrary time series input sequence such as non-segmented handwriting recognition, speech recognition, etc. Long short term memory (LSTM)^12^, as a variant of RNN, can effectively prevent the occurrence of gradient vanishing from processing time series signals. In recent years, remarkable achievements have been made in the field of pattern classification via the use of convolutional neural networks (CNNs) as deep learning structures^13–16^. CNNs provide an end-to-end learning model. The trained CNNs by the gradient descent method can learn the characteristics of input data and further complete the pattern classification. CNNs have strong ability of feature learning and pattern classification. The main reason is that the features of the lower layers are derived from the partial information and convolution kernel with sharing weights from the upper layer. CNNs have been applied in the classification of human physiological signal patterns. Based on a 34-layer CNN, Rajpurkar *et al*. classified the electrocardiogram (ECG) signals into 14 types^17^. Moreover, Rubin *et al*. performed heart-sound recordings based on deep CNN and Mel-frequency cepstral coefficients^18^. These studies used CNN to achieve pattern classification of relevant physiological signals and achieved higher accuracy than the diagnostic results of experienced physicians. Furthermore, Hu *et al*. used CNN to divide pulse waves into two types: health and subhealth^19^. In the present study, in view of the large amount of pathological and physiological information contained in pulse signals, we collected the required data under the guidance of the doctor and established two data sets based on either CVD/complication categories or physiological parameters. We proposed an optimised CNN model for PWPC based on these two data sets. The purpose of this study was to identify a practical and efficient classification criterion for PWPC based on CNN, which contributed to non-invasive, practical and effective diagnosis of CVDs and related complications.

## Results

**Figure 2:**
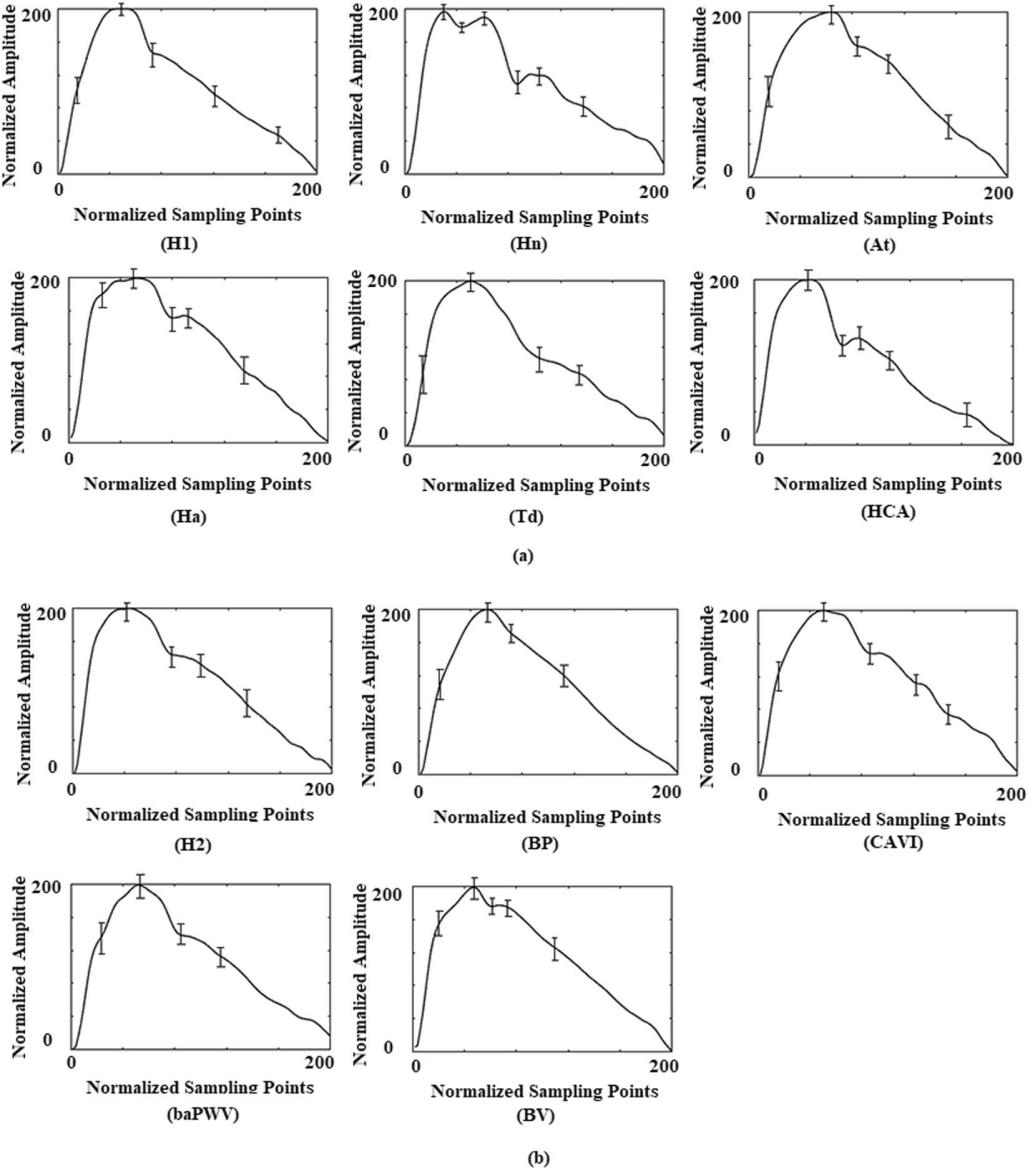
Average pulse-wave patterns in data set 1 (**a**) and data set 2 (**b**). Abbreviation Represents Pulse Wave Patterns: H1-Healthy Control Group in Data Set 1; Hn- hypertension; At-atherosclerosis; Ha-hyperlipidaemia; Td-type 2 diabetes; HCA-Hypertension complicated by atherosclerosis; H2-Healthy Control Group in Data Set 2; BP-blood pressure; baPWV- brachial-ankle pulse wave velocity; BV-blood viscosity.

The average pulse waves of each pattern in the two data sets are shown in Fig. 2.

**Figure 3:**
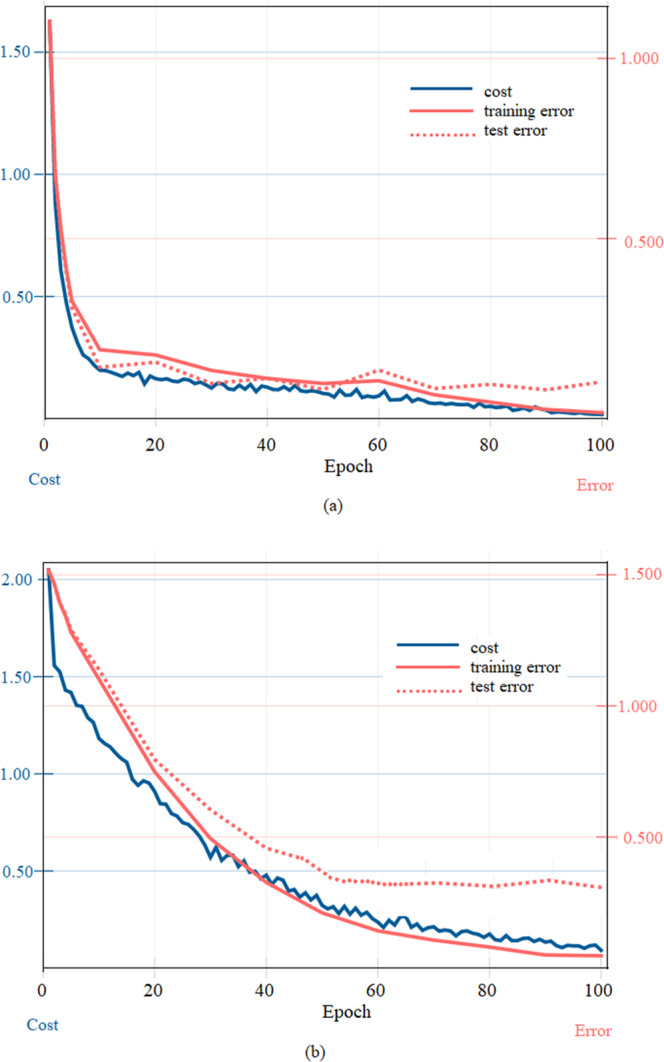
Learning curve in data set 1 (**a**) and data set 2 (**b**).

We showed the learning curves of data set 1 and data set 2 respectively to evaluate their PWPC performance on the proposed CNN model, as shown in Fig. 3. For cost-value curve, the decline rate of data set 1 was significantly higher than that of data set 2. For training error and test error, the minimum value of data set 1 was smaller than that of data set 2. Especially test error, data set 1 (When epoch was 90, the minimum test error was 0.08. Epoch was the number of iterations in CNN pattern classification) was much smaller than data set 2 (When epoch was 100, the minimum test error was 0.34). With the same proposed CNN, the six pulse patterns in data set 1 showed higher calculation efficiency and feature expression ability than those five patterns in data set 2.

Table 1 shows the overall values of the evaluation parameters in the two data sets. The accuracy and other evaluation parameters of PWPC in data set 1 (overall accuracy = 0.95) were higher than those in data set 2 (overall accuracy = 0.89). Tables 2 and 3 show the details for each pattern in the two data sets separately. Pulse-wave patterns representing healthy subjects (H1 and H2) could be identified with high precision (precision H1 = 1, recall H1 = 0.99; precision H2 = 0.97, recall H2 = 0.97). The HCA, as the pulse pattern of complications, had the lowest classification rate in data set 1 (precision HCA = 0.89, recall HCA = 0.91). In addition, the classification performance of other pulse patterns in data set 1 was higher than that in data set 2.

**Table 1:** PWPC evaluation of per pulse patterns in two data sets.

Data set	Overall accuracy	Overall precision	Overall recall	Overall F-measure
Data set 1	0.95	0.95	0.95	0.95
Data set 2	0.89	0.89	0.89	0.89

**Table 2:** PWPC evaluation of per pulse patterns in data set 1.

Pulse pattern	Precision	Recall	F-measure
H1	1	0.99	0.99
Hn	0.94	0.93	0.94
At	0.90	0.94	0.92
Ha	1	0.99	0.99
Td	0.96	0.93	0.94
HCA	0.89	0.91	0.90

**Table 3:** PWPC evaluation of per pulse patterns in data set 2.

Pulse pattern	Precision	Recall	F-measure
H2	0.97	0.97	0.97
BP	0.89	0.89	0.89
CAVI	0.82	0.84	0.83
baPWV	0.84	0.87	0.85
BV	0.95	0.89	0.92

To further assess the PWPC result of the proposed CNN model, the two data sets were put into different neural networks models for PWPC. Table 4 shows the accuracy of PWPC with those different models. It details network methods, classification criteria, number of subjects, and the accuracy. It demonstrated that compared with other neural networks or other CNN structures, our proposed CNN model achieved higher accuracy in PWPC under the new classification criteria, which also meant stronger feature extraction ability for pulse signals.

**Table 4:** PWPC accuracy of different methods.

Network	Method	Classification criteria	Number of subjects	Accuracy
The proposed CNN model	CNN	CVD and complications	412	**0.95**
	CNN	Physiological parameters	412	0.89
LetNet^38^	CNN	CVD and complications	412	0.69
	CNN	Physiological parameters	412	0.63
AlexNet^14^	CNN	CVD and complications	412	0.73
	CNN	Physiological parameters	412	0.70
VGG-Net^15^	CNN	CVD and complications	412	0.81
	CNN	Physiological parameters	412	0.79
Wang’s network^7^	Bayesian Network	Based on TCPD	407	0.84
Xu’s network^8^	Fuzzy Neural Network	Based on TCPD	320	0.90

**Figure 4:**
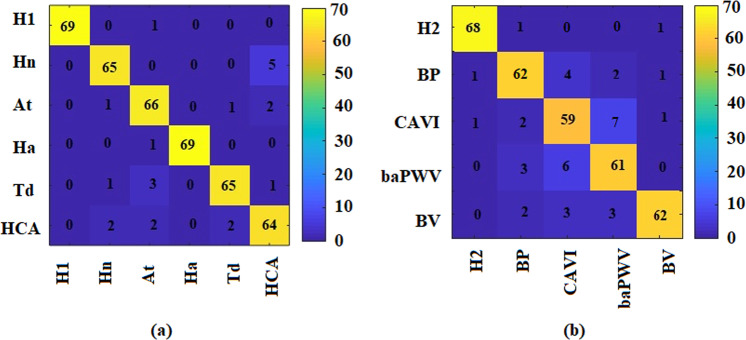
The confusion matrices of data set 1 (**a**) and data set 2 (**b**). The confusion matrix is an intuitive method for evaluating the results of pattern classification CNN models. The real categories (rows) and predicted categories (columns) of the classification results can be read directly. For example, in matrix (**a**), there were 70 (65 + 5) pulse waves which really belonged to the Hn pattern (the second row), while the CNN model predicted 69 (65 + 1 + 1 + 2) pulse waves in the Hn pattern (the second column).

To further analyse the causes of errors in pattern classification, we determined the confusion matrix of the two data sets, as shown in Fig. 4. The cause of errors in data set 1 was mainly the erroneous classification of the four pulse patterns of Hn, At, HCA and Td. In data set 2, with the exception of the control group (H2), the remaining four pulse patterns (BP, CAVI, baPWV and BV) were found to interfere with each other and have higher error rates.

## Discussion

In this study, CVD and associated complications as well as related physiological parameters were extracted, which were used as classification criteria. According to the new classification criteria, we screened the subjects’ pulse waves and created data set 1 and data set 2, respectively. An optimised CNN model was proposed for PWPC. It achieved the classification of six pulse patterns in data set 1 with an accuracy of 95% and the classification of six pulse patterns in data set 2 with an accuracy of 89%. The main contributions of this study are as follows:

1. Two pulse wave data sets were created, which contained a large amount of physiological and pathological information of subjects.
2. New classification criteria and optimized CNN model were proposed, which achieves higher accuracy than previous studies^7,8,19–21^.

This study demonstrates that CVD and complications are practical and efficient classification criteria, enabling the optimised CNN model to achieve high accuracy for PWPC.

We observed that the classification errors in data set 1 were mainly due to the erroneous classification of the Hn, At and HCA patterns. This was due to the simultaneous occurrence of hypertension and atherosclerosis on behalf of HCA. There must be some similar pulse characteristics between HCA and the other two diseases, which indicates that, in order to ensure that the characteristics of the different pulse patterns are typical, the selected data specimen must exclude the effect of complications at the same time. In addition, in data set 1, Td was also partially misclassified as Hn (n = 1), At (n = 3) and HCA (n = 1). Previous studies showed that type 2 diabetes could increase the risk and mortality of CVD, and they had similarities in the damage to the cardiovascular system^22–24^. Thus, there might have been similar pulse waveform characteristics between Td and Hn, At, HCA patterns, which led to classification errors.

**Figure 5:**
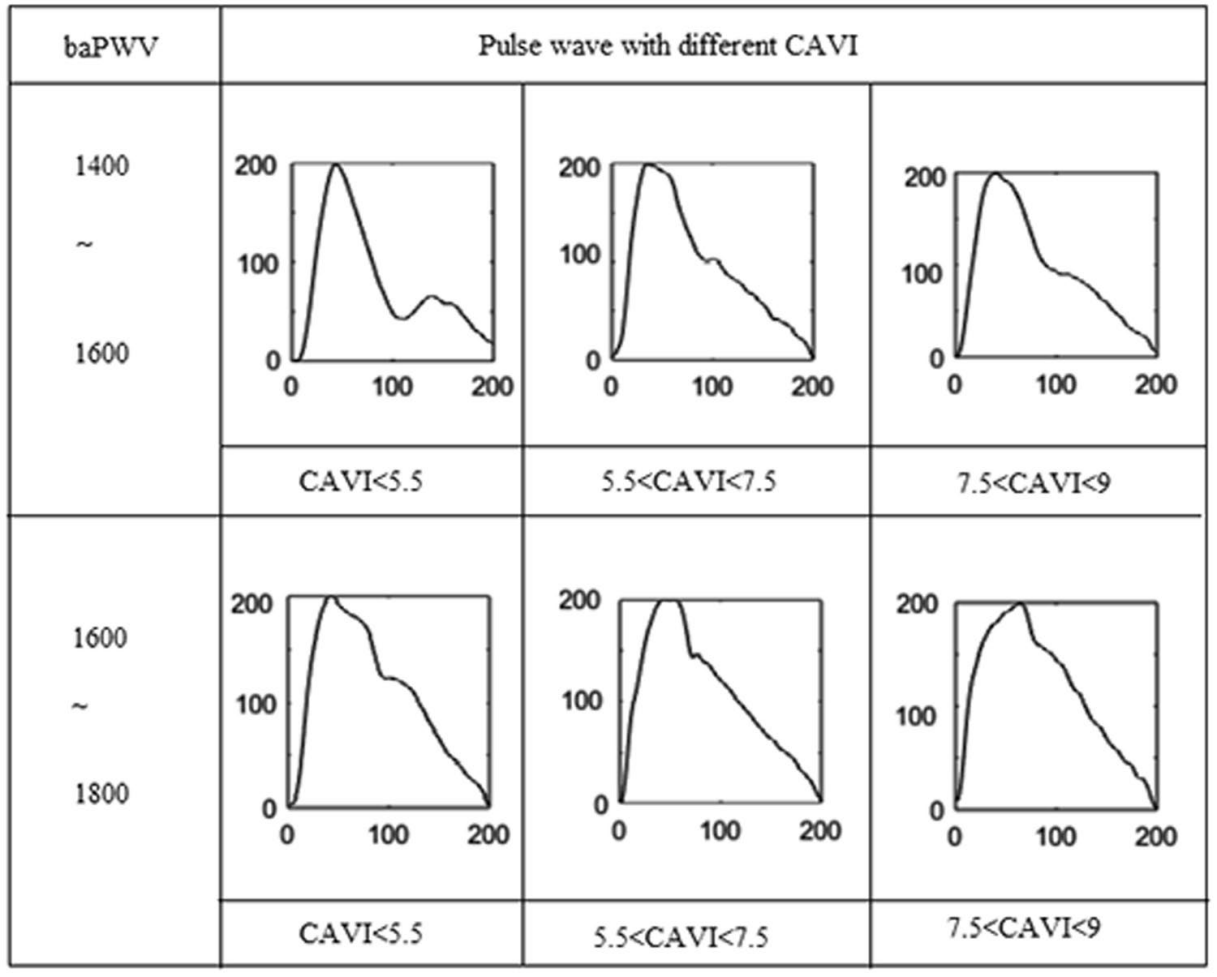
The pulse-wave form baPWV pulse pattern with the different baPWV and different CAVI. Pulse waves from six subjects were selected.

In data set 2, four pulse patterns (BP, CAVI, baPWV and BV) were found to interfere with each other in pattern classification. Previous studies showed that the effect of a single physiological parameter on pulse waveform was mainly reflected in the change of some local characteristics^25–27^. The pulse waveform characteristics with the same value of one specific physiological parameter would change as a result of the differences of other physiological parameters, as shown in Fig. 5. It may have led to the errors of pattern classification in data set 2. Our study showed that the pulse-wave was the result of multiple physiological parameters. There are clearly limitations associated with using a single physiological parameter in characterising the overall pulse pattern. Disease was the result of multiple physiological parameters, which might explain the higher classification accuracy in data set 1.

This study had several limitations. The most important one was the relatively limited number of subjects. Limited by the number of subjects, the effects of some physiological information such as age, height and weight on pulse waveform were ignored, which inevitably led to errors in pattern classification^1^. However, in our study, the number of pulse waves in each pulse pattern was several times that in some previous studies^21,28^. To some extent, the findings indicated that each of our patterns could represent the typical pulse characteristics. In addition, this study focused on the classification criteria of pulse patterns. For this purpose, we used the same CNN model to classify two data sets. Regarding the low classification rate of data set 2, we did not explore whether it could be improved by optimising the architecture of the CNN model.

## Conclusions

In this study, we established pulse wave data set 1 and data set 2 based on the classification criteria: CVD categories and related physiological parameters. CNN was used to extract features from two data sets and to achieve PWPC with high accuracy. The main contribution of this study is to propose the new classification criteria for PWPC and construct a matching CNN model. The optimized CNN model achieved PWPC with 95% accuracy in data set 1 and 89% accuracy in data set 2. This study demonstrated that pulse waves are the result of multiple physiological parameters, so there are limitations when using a single physiological parameter to characterize the overall pulse pattern. The proposed CNN model can achieve high accuracy PWPC while using CVD and complication categories as classification criteria, which contributes to non-invasive, practical and effective diagnosis of CVD and associated complications.

## Method

### Data collection

The original pulse wave data were from the “Study on Evaluation Method of Cardiovascular System Based on Non-invasive Detection of Blood Pressure and Pulse-Wave of Limbs^29^”, which recruited 412 subjects and determined their physiological parameters and more than 12,000 cycles of pulse waves. The pulse and blood pressure signal measuring device was Fukuda VS-1500A. In addition, the subjects’ brachial ankle pulse-wave velocity (baPWV) and blood viscosity were collected. All subjects were registered at Beijing University of Technology Hospital, and information on their diseases was collected through the subjects’ medical records.

The study with its experimental protocols and relevant details was approved by the Institutional Ethics Committee of Beijing University of Technology and Tohoku University. All experiments were performed in accordance with relevant guidelines and regulations. We explained the content of the study to the subjects in detail, and on this basis, the subjects signed the informed consent form.

### Pulse waveform denoising and normalisation

In this study, we collected the pulse signals from the wrist of the subjects. The denoising and normalization of pulse signals were processed with the same method as the previous studies^30^. Firstly, the noise was removed with wavelet transform decomposition method^31^. Then, in order to prevent the distortion of pulse signals, according to Nyquist theorem and actual sampling frequency^8,19^, the sampling points of single cycle of pulse wave were set at 200. Because the focus of this study was the change of pulse wave model, the amplitude of pulse wave was normalized to 0–200 in each cycle.

### Data sets

**Figure 6:**
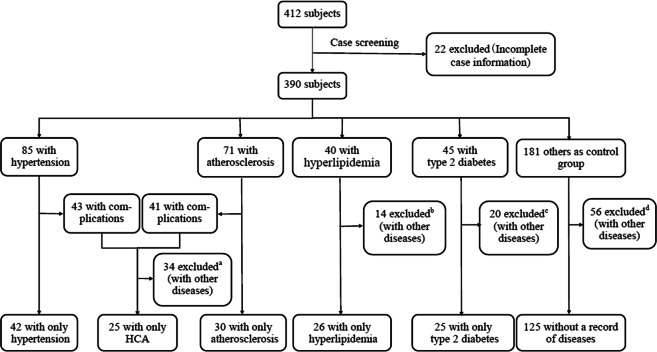
The process of screening the subjects in data set 1. ^a,b,c,d^Screening criteria: The number of subjects for a selected disease should be more than 20. The disease or complications must be of the five types selected in this study. There is no serious abnormality in pulse waves caused by noise or incorrect data collection, among others. We show all cases and numbers of excluded subjects in ^c^: type 2 diabetes complicated by hypertension (n = 4), type 2 diabetes complicated by atherosclerosis (n = 3), type 2 diabetes complicated by heart failure (n = 5) and diabetic foot disease (n = 8). Based on the screening criteria, we excluded these cases.

Previous studies classified pulses into patterns based on the TCPD theory^7–10,32^. However, as mentioned previously, under this classification criterion, one pulse pattern may correspond to a variety of disease categories. Thus, in this study, based on subjects’ clinical data, we directly selected five diseases as new classification criteria: hypertension, atherosclerosis, hyperlipidaemia, type 2 diabetes and hypertension complicated by atherosclerosis (HCA). Type 2 diabetes, as one of the common complications of CVD^33^, and HCA were used to study the effects of CVD complications on pulse waves. To ensure the typical characteristics of each pulse pattern, the pulse signals from subjects who only suffered from one of the five diseases and healthy subjects (a total of six types) were used as new pulse patterns to build data set 1, as shown in Fig. 6.

**Figure 7:**
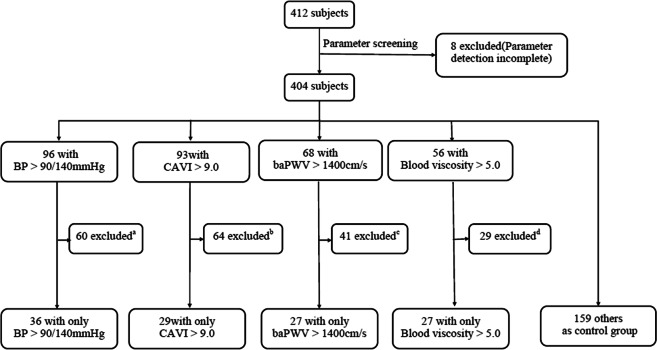
The screening process of the subjects in data set 2. ^a,b,c,d^Screening criteria: The number of subjects for selected parameters should be more than 20. Subjects’ other parameters, such as stroke output and cardiac output, must be within the normal range of medical reference. There is no serious abnormality in pulse-wave caused by noise or incorrect data collection, among others. For ^a,b,c,d^, most of the excluded subjects had three or even four parameter values outside of the range. To ensure that the characteristics of each pulse pattern were typical, we excluded these subjects.

We simultaneously selected four physiological parameters closely related to the selected diseases as classification criteria: blood pressure, which can be used as an indicator for assessing hypertension^34^; cardio-ankle vascular index (CAVI), which is one of the indicators for assessing atherosclerosis^35^; and brachial ankle pulse-wave velocity (baPWV), which can be used as an indicator for evaluating cardiovascular function in type 2 diabetics^36^; For patients with hyperlipidaemia, an increase of blood lipids often occurs simultaneously with increased blood viscosity^37^. Based on the subjects in data set 2 and the medical reference range, we determined the range of each physiological parameter. The pulse waves of subjects in whom only one of the four parameters was beyond the range were selected. The pulse waves of subjects whose four parameters were all within the range were also selected as a healthy control group. Then the five types of pulse pattern were used to build data set 2, as shown in Fig. 7.

For the processing of pulse image, this study used the same method as previous studies^30^. We extracted the pulse cycles from the selected subjects. To avoid data duplication affecting the accuracy of CNN prediction, all pulse waves in the two data sets were taken from different cycles. The total cycles of each pulse pattern were 210, which were divided into training set and test set, as shown in Table 5. As mentioned above, the number of sampling points in a single cycle of normalized pulse wave was 200, and the amplitude was 0–200. Therefore, the pulse wave signals were processed as input PNG pulse images with a size of 200 × 200 pixels.

**Table 5:** The details of PWPC data sets.

	Pulse categories	Total pulse number	Training number	Test number	Type of disease or physiological parameters (range)
Data set 1	H1	210	140	70	Healthy control group in data set 1
	Hn	210	140	70	Only hypertension
	At	210	140	70	Only atherosclerosis
	Ha	210	140	70	Only hyperlipidaemia
	Td	210	140	70	Only type 2 diabetes
	HCA	210	140	70	Hypertension complicated by atherosclerosis
Data set 2	H2	210	140	70	Healthy control group in data set 2
	BP	210	140	70	Only high blood pressure (>90/140 mmHg)
	CAVI	210	140	70	Only CAVI (>9.0)
	baPWV	210	140	70	Only baPWV (>1400 cm/s)
	BV	210	140	70	Only blood viscosity (>5.0)

### The proposed CNN

**Figure 8:**
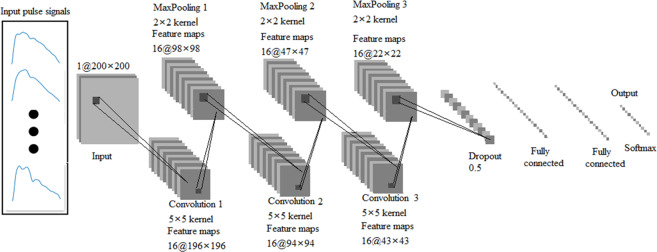
An illustration of the CNN architecture. The size settings of convolution kernels and feature maps are shown in the figure.

In this study, an optimised CNN model (10-layer) was proposed based on DCNN^19^ and LeNet-5^38^, which had been applied for PWPC, as shown in Fig. 8. Compared with the previous networks, we added dropout^39^ between the third max pooling layer and the fully connected layer. When CVDs were used as classification criteria, each pulse pattern changed from local waveform difference under previous criteria to overall pulse waveform difference. This led to too many characteristic parameters of pulse wave extracted by CNN, which further led to over-fitting in the training process. Pre-experimental results showed that dropout layer could help reduce test errors and avoid over-fitting phenomenon in the training process (see Supplementary Fig. S1). In addition, the final Softmax activation produced a distribution over the output probability classes for each pulse pattern of two data sets. Besides the layers mentioned above, the CNN also included three convolution layers, three max pooling layers and two fully connected layers. The number of convolution layers was determined by the number of pulse wave characteristic. The insufficient layers led to the inadequate feature extraction ability of CNN, while the excessive layers increased the time cost and calculation cost. In this study, we determined the number of layers by pre-experimental results. The convolutional layers were used to extract complex parameters of the input feature maps by convolution with kernels. The max pooling layers achieved the down-sampling of the input signals by choosing the maximum value of the area as the value of the pooled area. The max pooling layers could retain the main features of the input signals while reducing the parameters and computation, which helped to avoid the occurrence of over-fitting and improve the generalization ability of the CNN model^40^. The final two fully connected layers combined all of the upper feature maps into a one-dimensional array, which was used to classify the output. In this study, we used the Adam optimiser, which is straightforward to implement, with high calculation efficiency and low memory requirements^41^. In accordance with previous studies and a preliminary experiment, the parameters of the Adam optimiser were as follows: learning rate = 0.001, ϵ = 0.001, ρ1 = 0.9, ρ2 = 0.999 and δ = 1E^−8^. During the optimisation process, we saved the best model configuration as evaluated on the test set. The CNN was trained by neural_network_console (Sony Company) on an Intel(R) HD Graphics 630 with batch size 64 for 100 epochs.

### Evaluation

The proposed CNN was evaluated with the average of the operating parameters calculated over time. The overall accuracy, precision, recall and F-measure were determined to assess the classification performance of the network, as presented in the results section. To further evaluate the classification performance of each pulse pattern, we also present the evaluation parameters of each pattern and the confusion matrices for the two test sets. The evaluation parameters were calculated using the true positive (TP), true negative (TN), false positive (FP) and false negative (FN).

In order to further evaluate the PWPC capability of the CNN model proposed in this study, we selected three different neural networks (LetNet^38^, AlexNet^14^, VGG-Net^15^). Data set 1 and data set 2 were used as inputs of these three networks respectively. The PWPC results were compared with the CNN model proposed in this study.

### Supplementary information

Supplementary Information

## Data Availability

The datasets analysed during the current study are available from the corresponding author on reasonable request.
