# Author Correction: Pulse-Wave-Pattern Classification with a Convolutional Neural Network

**DOI:** 10.1038/s41598-024-57035-9

**Published:** 2024-03-26

**Authors:** Gaoyang Li, Kazuhiro Watanabe, Hitomi Anzai, Xiaorui Song, Aike Qiao, Makoto Ohta

**Affiliations:** 1 Institute of Fluid Science, Tohoku University, 2-1-1, Katahira, Aoba-ku, Sendai, Miyagi 980-8577 Japan; 2 Graduate School of Biomedical Engineering, Tohoku University, 6-6 Aramaki-aza-aoba, Aoba-ku, Sendai, Miyagi 980-8579 Japan; 3 Department of Radiology, Taishan Medical University, No.619 Greatwall Road, Daiyue District, Taian, 271000 Shandong China; 4 College of Life Science and Bioengineering, Beijing University of Technology, No.100, Pingleyuan, Chaoyang District, Beijing, 100022 China; 5 ELyTMaX UMI 3757, CNRS–Université de Lyon–Tohoku University, Sendai, Japan

Correction to: *Scientific Reports* 10.1038/s41598-019-51334-2, published online 17 October 2019

This Article contains errors in the Data availability statement.

The Data availability statement should read: “Anonymised versions of the datasets analysed during the current study are available upon request to Prof Aike Qiao (qak@bjut.edu.cn) and with a signed agreement from Beijing University of Technology.”

