# Supplementary material for: Pulse-Wave-Pattern Classification with a Convolutional Neural Network
Source: Sci Rep. 2019 Oct 17;9:14930. doi: 10.1038/s41598-019-51334-2 (PMC6797811; doi:10.1038/s41598-019-51334-2)
Supplement: Supplementary file 1 — Supplementary Information [file 41598_2019_51334_MOESM1_ESM.docx]

**Supplementary Information:**

**Pulse-Wave-Pattern Classification with a Convolutional Neural Network**

**Gaoyang Li^1,2^, Kazuhiro Watanabe^1,2^, Hitomi Anzai^2^, Xiaorui Song^3^, Aike Qiao^4^, Makoto Ohta^2,5*^**

^1^Institute of Fluid Science, Tohoku University, 2-1-1, Katahira, Aoba-ku, Sendai, Miyagi ,980-8577, Japan

^2^Graduate School of Biomedical Engineering, Tohoku University, 6-6 Aramaki-aza-aoba, Aoba-ku, Sendai, Miyagi ,980-8579, Japan

^3^Department of Radiology, Taishan Medical University, No.619 Greatwall Road, Daiyue District, Taian, Shandong, 271000, China

^4^College of Life Science and Bioengineering, Beijing University of Technology, No.100, Pingleyuan, Chaoyang District, Beijing, 100022, China

^5^ELyTMaX UMI 3757, CNRS–Université de Lyon–Tohoku University

^*^Corresponding author: [makoto.ohta@tohoku.ac.jp](mailto:makoto.ohta@tohoku.ac.jp)

# Additional detail on Dropout layer

As shown in the supplementary Figure 1 below, we show the learning curve (data set 1) with (a) and without (b) dropout layer of pre-experiment. If dropout layer is not added, it can be found that the rate of decline of error curve is obviously slower. And there is overfitting phenomenon (Test errors begin to increase, which means accuracy begin to decrease). The pre-experiment results proved the necessity of adding Dropout layer in CNN network proposed by this study.


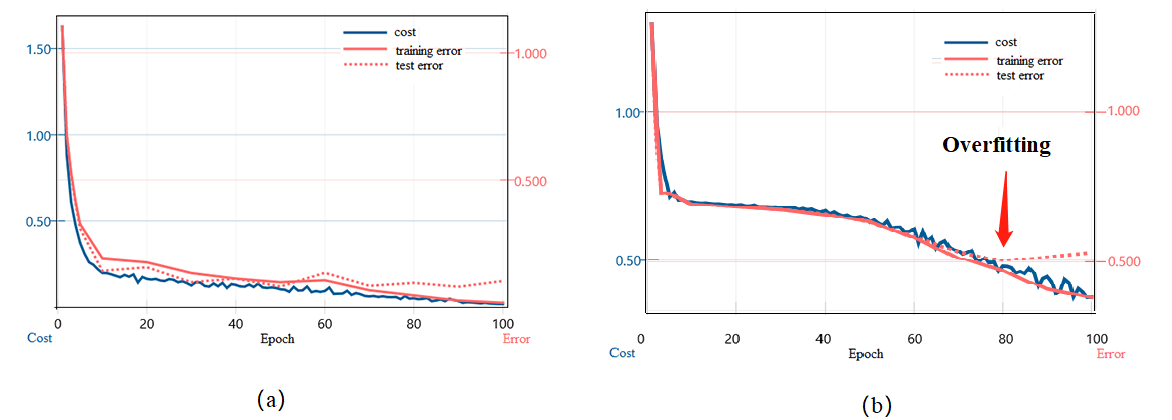


**Supplementary Figure 1.** Learning curve (data set 1) with (a) and without (b) dropout layer. For (a), the decline rate of cost curve is significantly higher than that of (b). At the same time, the final test error value of (a) is obviously less than that of (b), which means that over-fitting phenomenon occurs.
